# Mammalian-Specific Sequences in *Pou3f2* Contribute to Maternal Behavior

**DOI:** 10.1093/gbe/evu072

**Published:** 2014-04-07

**Authors:** Makoto Nasu, Saori Yada, Atsushi Igarashi, Den’etsu Sutoo, Kayo Akiyama, Meguru Ito, Nobuaki Yoshida, Shintaroh Ueda

**Affiliations:** ^1^Department of Biological Sciences, Graduate School of Science, The University of Tokyo, Japan; ^2^Institute of Medical Science, University of Tsukuba, Japan; ^3^Center for Experimental Medicine and Systems Biology, Institute of Medical Science, The University of Tokyo, Japan

**Keywords:** nonmammalized mice, amino acid repeats, transcription factor, *Pou3f2*/*Brn-2*, maternal behavior

## Abstract

Various mutations have occurred during evolution among orthologs, genes in different species that diverged from a common ancestral gene by speciation. Here, we report the remarkable deterioration of a characteristic mammalian maternal behavior, pup retrieval, in nonmammalized mice, in which the transcription factor *Pou3f2* was replaced with the *Xenopus* ortholog lacking all of the homopolymeric amino acid repeats of mammalian POU3F2. Most of the pups born to the nonmammalized mice died within days after birth, depending on the dam genotype alone. Quantitative immunohistochemical analysis revealed decreases in the rate-limiting enzymes of dopamine and serotonin synthesis in various brain structures. Similar results were obtained in knock-in mice in which all of the homopolymeric amino acid repeats of mammalian POU3F2 were removed. Pup retrieval behavior in mammals is thus strongly related to monoamine neurotransmitter levels via the acquisition of homopolymeric amino acid repeats during mammalian evolution.

## Introduction

Organisms belonging to a particular taxon have unique features that can be distinguished from those belonging to other taxa. For mammals, the typical features are hair, three middle ear bones, and a neocortex. The word “mammal” originates from the word “teat”, highlighting the feature that all mammalian females nurse their pups with milk. Recent progress in DNA sequencing technology has made whole-genome sequences available from a wide variety of vertebrates. Genome-wide comparisons have revealed characteristics of the evolutionary change of different aspects of genomes involving chromosomes, genes, regulatory regions, and retrotransposons. A major difference between mammals and other vertebrates is the presence or absence of homopolymeric amino acid repeats (sequences without interruptions in the run of a single amino acid residue), indicating that these repeats were acquired specifically in the mammalian lineage (Sumiyama et al. 1996; Nakachi et al. 1997; Albà and Guigó 2004; Faux et al. 2005). This remarkable feature is well conserved in both position and repeat number among mammals. Some types of homopolymeric amino acid repeats modulate protein–protein interactions and/or transcriptional regulation, and cause phenotypic diversification (Gerber et al. 1994; Xiao and Jeang 1998; Fondon and Garner 2004; Anan et al. 2007).

Transcription factor *Pou3f2/Brn-2* is prominently expressed in the neocortex. The neocortex has undergone pronounced expansion and development during evolution, and is considered to be responsible for cognitive function, sensory perception, and consciousness. The mammalian neocortex comprises a highly organized six-layered structure, with each unique layer containing neurons having similar morphologies and projection patterns. Compared with nonmammalian vertebrates, the upper layers (II–IV) are the most prominent distinguishing feature of mammalian neocortex. *Pou3f2* is expressed primarily in neurons of layers II–V (He et al. 1989) and is involved in cortical neural migration (McEvilly et al. 2002), layer production (Sugitani et al. 2002), and neurogenesis (Castro et al. 2006). Cortical neurons that comprise each layer of the neocortex are generated in the ventricular zone, where *Pou3f2* expression is detected (Alvarez-Bolado et al. 1995). Forced expression of *Pou3f2* with a combination of two transcription factors, *Ascl-1*/*Mash-1* and *Myt1-l*, can convert fibroblasts into functional neurons (Vierbuchen et al. 2010; Pang et al. 2011; Pfisterer et al. 2011).

Mammalian POU3F2 has glycine, glutamine, and proline repeats, whereas most or all of these repeats are absent from POU3F2 orthologs in nonmammalian vertebrates (Sumiyama et al. 1996; Nakachi et al. 1997). To trace back mammalian characteristics, we investigated the changes that may occur in the presence or absence of homopolymeric amino acid repeats. Here, we report our discovery that mice with a nonmammalized *Pou3f2* gene show less curiosity toward their pups, drastically decreased pup retrieval behavior that leads to increased pup death, and decreased monoamine neurotransmitter production in the brain.

## Materials and Methods

All living modified organisms and animal experiments were approved by The University of Tokyo, and conducted in accordance with the guidelines. Abbreviations: wild-type, *+/+*; *xPou3f2* knock-in heterozygous, *+/tro*; *Pou3f2 ΔGQP* knock-in heterozygous, *+/Δ*; *xPou3f2* knock-in homozygous, *tro/tro*; and *Pou3f2 ΔGQP* knock-in homozygous, *Δ/Δ*.

### Multiple Alignment

POU3F2 amino acid sequences were obtained via translation from transcript sequences registered in DDBJ/EMBL/GenBank. The sequence IDs are given in supplementary table S1, Supplementary Material online. To create multiple alignments, we used MAFFT on a CLC Bio Sequence Viewer (http://www.clcbio.com/, last accessed May 1, 2014).

### Targeting Vector Construction

**Fig. 1.—:**
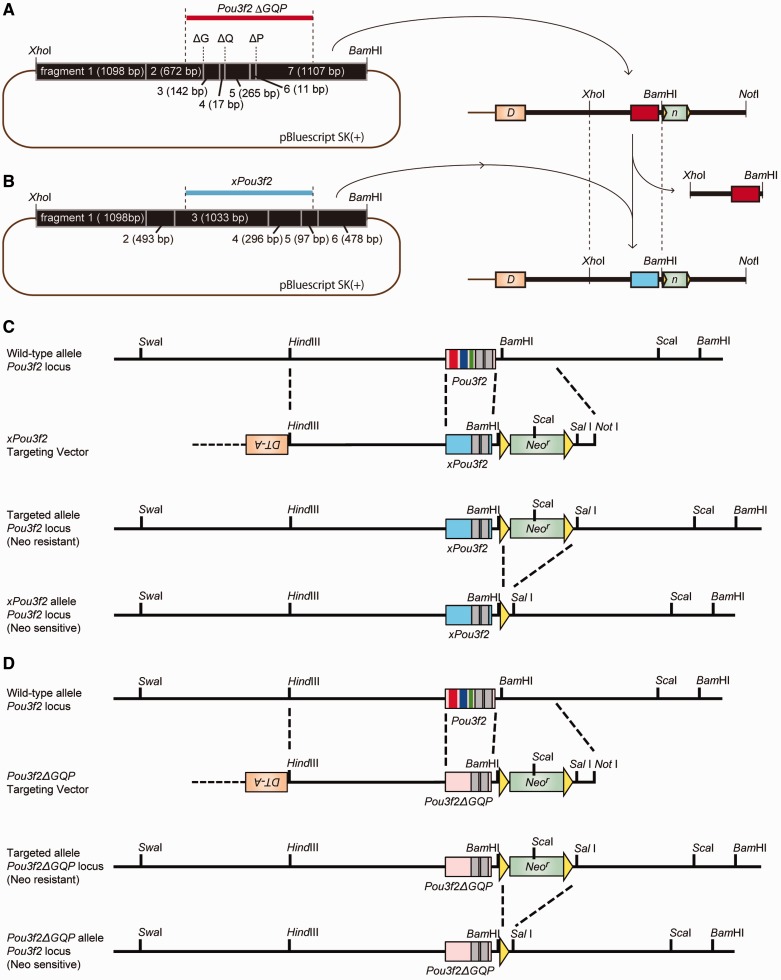
Targeting vector construction and targeting strategy. (*A*) Mouse genome fragment (black box) from *Xho*I to *Bam*HI, including *Pou3f2 ΔGQP* (red) in pBluescript SK(+) (left). Targeting vector was created using this fragment (right). Green box is *Neo^r^*, orange box is *DT-A*, bold black line is mouse genome, and brown line is pBluescript SK(+). *DT-A*, diphtheria toxin fragment A; *Neo^r^*, neomycin-resistance gene. (*B*) Mouse genome fragment from *Xho*I to *Bam*HI including *xPou3f2* (blue) in pBluescript SK(+) (left). This fragment was used for the targeting sequence (right). (*C*) Strategy for generating the *xPou3f2* knock-in mouse. (*D*) Strategy for generating the *Pou3f2 ΔGQP* knock-in mouse. Yellow triangles indicate the sequence of *loxP* sites. Restriction sites are shown in the schematic sequence.

Amphibian genomic DNA was extracted from *Xenopus tropicalis* liver. Liver homogenates were incubated in 0.5 ml lysis buffer (10 mM Tris–HCl pH 8.0, 15 mM NaCl, 10 mM EDTA pH 8.0, and 0.1% sodium dodecyl sulfate) and 100 µg/ml proteinase K (Nakalai Tesque) at 55 °C overnight. RNase (50 µg/ml; Nakalai Tesque) was added to the samples. After incubation at 37 °C for 1 h, genomic DNA was purified by phenol–chloroform–isoamyl alcohol treatment and isopropanol precipitation. Using this DNA, the *xPou3f2* coding region (1,149 bp) was isolated by polymerase chain reaction (PCR) amplification (forward primer: 5′-GTCAAATGCTCGGCTCCTTTAAGC-3′ and reverse primer: 5′-CCCACTTTGGAAGTGGGATAGTGG-3′) and cloned using the TOPO TA Cloning Kit (Invitrogen, Carlsbad, CA, USA). *Pou3f2 ΔGQP* and *xPou3f2* were constructed according to the strategy shown in figure 1.

### Gene Targeting and Generation of *xPou3f2* and *Pou3f2 ΔGQP* Knock-in Mice

Each targeting vector was linearized at the *Not*I site and electroporated into 1.0 × 10^7^ embryonic stem cells derived from 129/SvOla E14.1. Embryonic stem cells were cultured on a feeder layer of primary cultured mouse embryonic fibroblasts in Dulbecco’s modified Eagle’s medium containing 15% heat-inactivated fetal calf serum, 7 × 10^−^^6 ^µl/ml 2-mercaptoethanol, and 2 µl/ml leukemia inhibitory factor to maintain pluripotency. After 24 h, neomycin-resistant clones were isolated by G418 (neomycin) selection at 20 mg/ml for 7 days. Neomycin resistance should be endowed by homologous recombination, replacement of mouse *Pou3f2* with *xPou3f2* (or *Pou3f2 ΔGQP*) and *neo^r^*. Eventually, some clones were identified as heterozygous for mouse *Pou3f2* and *xPou3f2* (or *Pou3f2 ΔGQP*) with *neo^r^* by Southern blot screening using a 5′-probe, 3′-probe, and neo probe. For Southern blot analysis, the genomic DNA was digested with *Sca*I and *Swa*I.

To remove *neo^r^*, a pIC-*Cre* vector including *Cre* recombinase was electroporated into a neomycin-resistant clone. pIC-*Cre* injected neomycin-sensitive clones were isolated by Southern blot screening using the neo probe. These homologous recombinated embryonic stem cells were verified by nucleotide sequencing. Two primer sets that cover the *Pou3f2* coding region were used—forward 1: 5′-GTAACTGTCAAATGCGCGGCTCCTTTAACC-3′, reverse 1: 5′-TTGCTGGTGTGGGTGAGAGTGCGGATG-3′, forward 2: 5′-CTCACCAGTGGATCACCGCGCTGTC-3′, reverse 2: 5′-CACCTGCTACCTGATATAGGAATAGTCC-3′.

Homologous recombinated clones were injected into C57BL/6 J blastocysts after treatment with HEPES. Chimeric male mice were mated with C57BL/6 J female mice and F1 (N1P1) mice were selected by PCR genotyping. In this study, F7 and later generation mice were used for analysis.

### Determination of Genotype

Genotyping was performed when the pups were 4 weeks old. We punched holes in the ears of mice to identify individual mice and cut the tips of their tails. The tail tips were dissolved in lysis buffer (5 M NaCl, 1 M Tris–HCl pH 8.0, and 0.5 M EDTA pH 8.0) with 1% sodium dodecyl sulfate, 1 µg/µl pronase E, and 0.1 µg/µl proteinase K at 55 °C overnight. The next day, the tail tips in lysis buffer were treated and purified with phenol, phenol–chloroform–isoamyl alcohol, and chloroform. Mouse genomic DNA was isolated for genotyping after isopropanol precipitation.

Genotyping primers were designed to amplify each three repeat region of mouse *Pou3f2*. The *xPou3f2* primers in polyQ and polyP regions were designed from mouse *Pou3f2* and *xPou3f2*, respectively. In PCR genotyping for polyQ and polyP regions, only primers derived from mice (mQ and mP) can bind to mouse *Pou3f2*, whereas only primers derived from amphibians (xQ and xP) can bind to *xPou3f2*. Therefore, primers derived from mouse and amphibians were mixed when amplifying polyQ or polyP regions. Primers for the polyG region were designed only from mouse *Pou3f2* because sequences around polyG are highly conserved between mice and amphibians. Expected amplification sizes were: G, 388 bp (mice)/328 bp (*X. tropicalis* and ΔG); Q, 414 bp (mice)/210 bp (*X. tropicalis*)/351 bp (ΔQ); and P, 370 bp (mice)/263 bp (*X. tropicalis*)/349 bp (ΔP).

Primers:

G-forward primer: 5′-GTAACTGTCAAATGCGCGGCTCCTTTAACC-3′,
G-reverse primer: 5′-GCTGTACCACCACCGAGGGCTTGATGTC-3′,
mQ-forward primer: 5′-ATCAAGCCCTCGGTGGTGGTACAGCAG-3′,
mQ-reverse primer: 5′-TTGCTGGTGTGGGTGAGAGTGCGGATG-3′,
xQ-forward primer: 5′-CAGGACATCAAGCCCTCAGT-3′,
xQ-reverse primer: 5′-AGTGAAGCTGGGCTGAGAGT-3′,
mP-forward primer: 5′-AGCAACAACAGCGACCGCCACATC-3′,
mP-reverse primer: 5′-AAACCAAACTCTCACCACCTCCTTCTCCAG-3′,
xP-forward primer: 5′-AGCCCAGCTTCACTGTCAAT-3′,
xP-reverse primer: 5′-AACGTCCGCCTCTCTAAATC-3′.

### Mice

All mice were housed under constant temperature (22–24 °C) and constant humidity (30–70%) conditions with a 12–12 h light–dark cycle. Food and water were provided ad libitum. Behavioral tests were performed in the behavioral test room where conditions were the same as above. Body weight was measured at postnatal day 70 (10 weeks of age). Virgin female mice (10–14 weeks of age) were used for the retrieval test, habituation–dishabituation test, and immunohistochemical analysis.

### Retrieval Test

A virgin female mouse (test mouse) was isolated in a test cage for 6 days to build a nest in a corner. Just before the retrieval test, this home cage was placed on a detector stand for 10–20 min to habituate the test mouse. When the test mouse had calmed down and remained at the nest, three wild-type (*+/+*) foster pups (postnatal days 1–6) were placed in each corner of the cage except for the nest corner. We defined each measurement item as follows: “first contact” was the contact with the nose of the test mouse to a pup, and “retrieval” was defined as the test mouse transferring a pup to the nest. Retrieval was recorded by video camera for 10 min. We measured the latency to the first contact, the latency to retrieve each pup, and the number of retrieved pups. The score for each pup that was not contacted and retrieved by the test mouse within 10 min was defined as 600 s.

### Habituation–Dishabituation Test

A virgin female mouse (test mouse) was habituated to an experimental cage for 20–25 min. A sexually immature young female mouse (*+/+*, 3–4 weeks of age, target mouse) was set in the feed box of this experimental cage. As this cage and the feed box were isolated by a filter paper, the test mouse in the cage could not directly contact the target mouse in the feed box, but could only sniff it.

After sufficient habituation, a target mouse “A” was placed into the feed box for 2 min. The target mouse was removed and after a 1-min interval, the same mouse “A” was again placed into the feed box. This was repeated four times (A1–A4) and for the fifth trial a different target mouse “B” was set into the feed box (B1). At the sixth trial, target mouse “A” was again placed into the feed box (A5). Each trial comprised 2-min contact and 1-min interval phases. In this study, “sniffing” was defined as contact with filter paper by the nose of the test mouse for more than 1 s. In general, mice have novelty-seeking behaviors. In this study, all tests were performed using virgin female mice. When a mouse showed no sniffing behavior midstream of the trial, the test was terminated. The number of completed test trials was as follows: *n* = 22 (A1), *n* = 22 (A2), *n* = 17 (A3), *n* = 14 (A4), *n* = 13 (B1), and *n* = 12 (A5) for*+/+*; *n* = 17 (A1), *n* = 16 (A2), *n* = 14 (A3), *n* = 13 (A4), *n* = 12 (B1), and *n* = 10 (A5) for *tro/tro*.

### Tissue Preparation

Mice were deeply anesthetized with pentobarbital and perfused through the heart with 15–20 ml of phosphate-buffered saline (PBS, pH 7.4) to remove the blood, and immediately followed by 50 ml of ice-cold fixative containing 4% paraformaldehyde and 0.2% glutaraldehyde in PBS. The animals were kept on crushed ice throughout the procedure. After perfusion, the whole brain was removed from the skull and postfixed with the same fixative solution at 4 °C for 2 h, then placed in 5% buffered sucrose at 4 °C overnight. Each brain block was embedded in Tissue-Tek O.C.T. Compound (Sakura Finetek Japan Co., Ltd) and frozen on liquid nitrogen and stored at −80 °C until use. Frozen tissue blocks were sectioned serially at a thickness of 20 µm in a cryostat HM505E (Carl Zeiss, Germany) at −20 °C, and sliced sections were mounted on 0.5% gelatin-coated glass slides. Brain slices located approximately 0.74 mm rostral from bregma and 4.60 mm caudal from bregma were used for tyrosine hydroxylase (TH) and tryptophan hydroxylase 2 (TPH2) immunohistochemistry, respectively.

### Immunohistochemistry

A polyclonal antibody to TH (catalog No. AB152, EMD Millipore Corporation, Billerica, MA, USA) and TPH2 (catalog No. ABN60, EMD Millipore Corporation) were used for immunohistochemistry as the primary antibody. Fluorescein isothiocyanate-labeled goat anti-rabbit IgG (catalog No. 65-6111, Invitrogen) was used as the secondary antibody. The immunohistochemical staining procedure was performed according to previously reported methods and conditions (Sutoo et al. 2002). Slices were rinsed with PBS four times for a total of 60 min. Primary antibodies were diluted 1:200 for TH and 1:400 for TPH2 with PBS and placed on each brain slice, then reacted for 12 h at 4 °C in a moist dark box. After reaction, the slices were rinsed four times with PBS for a total of 60 min. The secondary antibody was diluted 1:100 with PBS, placed on each slice, and incubated at 24 °C for 3 h in a moist dark box. After the reaction, the slices were rinsed four times with PBS for a total of 60 min. Finally, these slides were embedded in 10% glycerin in PBS and kept moist at 4 °C in a dark box.

### Distribution Analysis

The TH or TPH2 levels were quantitatively analyzed using a microphotometry system, which is a brain mapping analyzer (MapAnalyzer, Yamato Scientific Co., Ltd, Japan). The immunohistochemical distribution of each neurochemical in each brain region was examined at the cellular level throughout whole-brain slices as follows: 1) the target substance labeled by immunofluorescent staining in a microarea of a brain slice was illuminated by a fine excitation beam; 2) the fluorescence in this area was collected into the photometer, and its intensity measured; 3) the brain slice was moved by a two-dimensional scanning stage, and the next microarea was measured; and 4) the measured fluorescence intensity in each microarea was collected in a host computer, where it was analyzed for reconstruction of an image of the entire scanned area. The quantitative linearity, sensitivity, and resolution of this analyzer surpass those of image analyzers used with TV cameras by two orders of magnitude, and the sensitivity, reproducibility, and facility of this method are greater than those of the high-performance liquid chromatography method by at least three orders of magnitude (Sutoo et al. 1998, 2002). The same data can be obtained even after 50 measurements because the measuring point is irradiated for only 10 ms and thus fading of the fluorescence is negligible (Sutoo et al. 1998). The entire area of the slice was measured at 20 -µm intervals and approximately 130,000–200,000 data points were obtained. The operating conditions were as follows: excitation range, 465–495 nm; photomultiplier voltage, 800 V; objective, 20×/0.50 (magnification/numerical aperture); field diaphragm, 10 µm diameter; and photometry diaphragm, 40 µm diameter. The standard value of fluorescence intensity was calibrated using 1 mM quinine sulfate in 0.05 M sulfuric acid (100 µm in depth) (Sutoo et al. 1998), which is proportional to a fluorescence intensity of 100. After analysis, we selected 10 regions for TH and 10 regions for TPH2 for statistical analysis. The fluorescence intensity in each brain region was determined from both sides of two to four slices in each of seven to ten mice per genotype. Fluorescence intensity of TH in each region is indicated as a ratio to the mean intensity of four cortex regions (primary motor cortex, primary sensory cortex, dysgranular insular cortex, and piriform cortex), and that of TPH2 in each region is indicated as a ratio to the mean intensity of five control regions (external cortex of the inferior colliculus, mesencephalic reticular formation, lateral lemniscus, decussation of the superior cerebellar peduncle, and reticulotegmental nucleus of the pons). Student’s *t* test was used to compare *+/+* mice with *tro/tro* or *Δ/Δ* mice.

## Results and Discussion

### Most Pups of *tro/tro* Dams Died before Weaning

**Fig. 2.—:**
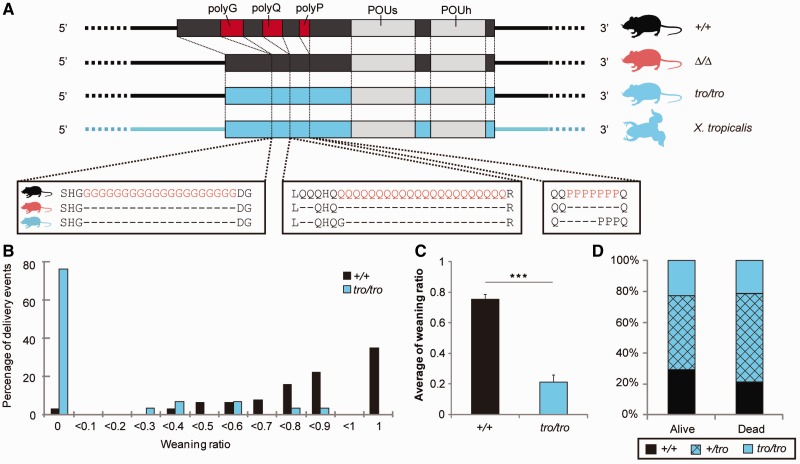
Weaning ratio. (*A*) Schematic of *Pou3f2* genomic region in *+/+* mice, two types of knock-in mice, and *Xenopus tropicalis*. Box shows *Pou3f2* coding region, and straight lines indicate *Pou3f2* upstream and downstream regions. Three homopolymeric amino acid repeats are shown in red, and DNA-binding domains are shown in gray. (*B*) Distribution of weaning ratio. Black and blue represent the weaning ratio of pups for *+/+* and *tro/tro* dams, respectively. (*C*) Mean weaning ratio. ***P < 0.001 (Student’s *t* test). (*D*) Genotype ratio of pups from *+/tro* parents.

To investigate the types of changes induced by inserting a nonmammalian *Pou3f2* gene into a living mammalian body, we newly generated knock-in mice, *xPou3f2* knock-in, in which the entire coding region of the murine *Pou3f2* gene was replaced with that of the amphibian (*X. tropicalis*) ortholog (vertebrate *Pou3f2* genes are intron-less) (figs. 1 and 2*A*, and supplementary fig. S1 and table S1, Supplementary Material online). We observed no change in appearance, body weight, growth, or reproductivity. Both homozygous (*tro/tro*) and heterozygous (*+/tro*) *xPou3f2* knock-in mice grew to adulthood and appeared normal. Both the expression pattern and the expression level of the mutant *tro* allele were similar to those of the wild-type allele (supplementary fig. S2*A*, Supplementary Material online). Body weight and brain weight per body weight did not differ between *+/+* and *tro/tro* mice (supplementary fig. S2*B* and *C*, Supplementary Material online). Further, no histologically abnormal changes were detected within the brain (supplementary fig. S2*D* and *E*, Supplementary Material online), whereas *Pou3f2* knockout and *Pou3f2*/*Pou3f3* double-knockout homozygous mice show severe brain abnormalities and die soon after birth (McEvilly et al. 2002; Sugitani et al. 2002). Nevertheless, most of the pups born to *tro/tro* dams could not develop to weaning, whereas most pups from *+/+* dams could (fig. 2*B*–*D*; statistically significant difference in weaning ratio compared with *+/+* pups, *P* < 0.001). Infanticide was not observed. If the pups had any genotypic defects causing the lower weaning ratio and viability, the weaning ratio of *tro/tro* pups born to *+/tro* parents would be expected to be lower than that of *+/tro* and *+/+* pups, and that of *+/tro* pups lower than that of *+/+* pups. The genotypic ratio of the pups obeyed Mendel’s law, however, regardless of successful or failed weaning. We therefore calculated the weaning ratio of pups born to *+/tro* dams mating with *+/+*, *+/tro*, and *tro/tro* sires. Nearly 65% of the pups failed to wean, and the values were independent of the genotype of the sires. In this study, the sires were removed just before delivery, and the dams nurtured the pups alone. Maternal behavior is critical for pup survival and these findings indicated that success or failure in weaning was due to the genotypes of dams and not that of the pups or sires. Moreover, the weaning ratio of the pups differed between the *+/+*, *+/tro*, and *tro/tro* dams (75.1%, 35.9%, and 20.7%, respectively), showing a clear regressive tendency toward a decreasing weaning ratio with a decrease in the number of nonmammalian *Pou3f2* alleles (*P* < 0.001, regression analysis). Nearly all of the pups that failed in weaning died within the first several days after birth. Parental care is essential for newborn mammals to survive until the weaning period (Rosenblatt and Lehrman 1963; Lerch-Haner et al. 2008). Our observations indicate that the low weaning ratio of pups was caused by defective maternal behavior. Therefore, we next focused on nurturing behaviors.

### Decreased Pup Retrieval by Nonmammalized Dams

**Fig. 3.—:**
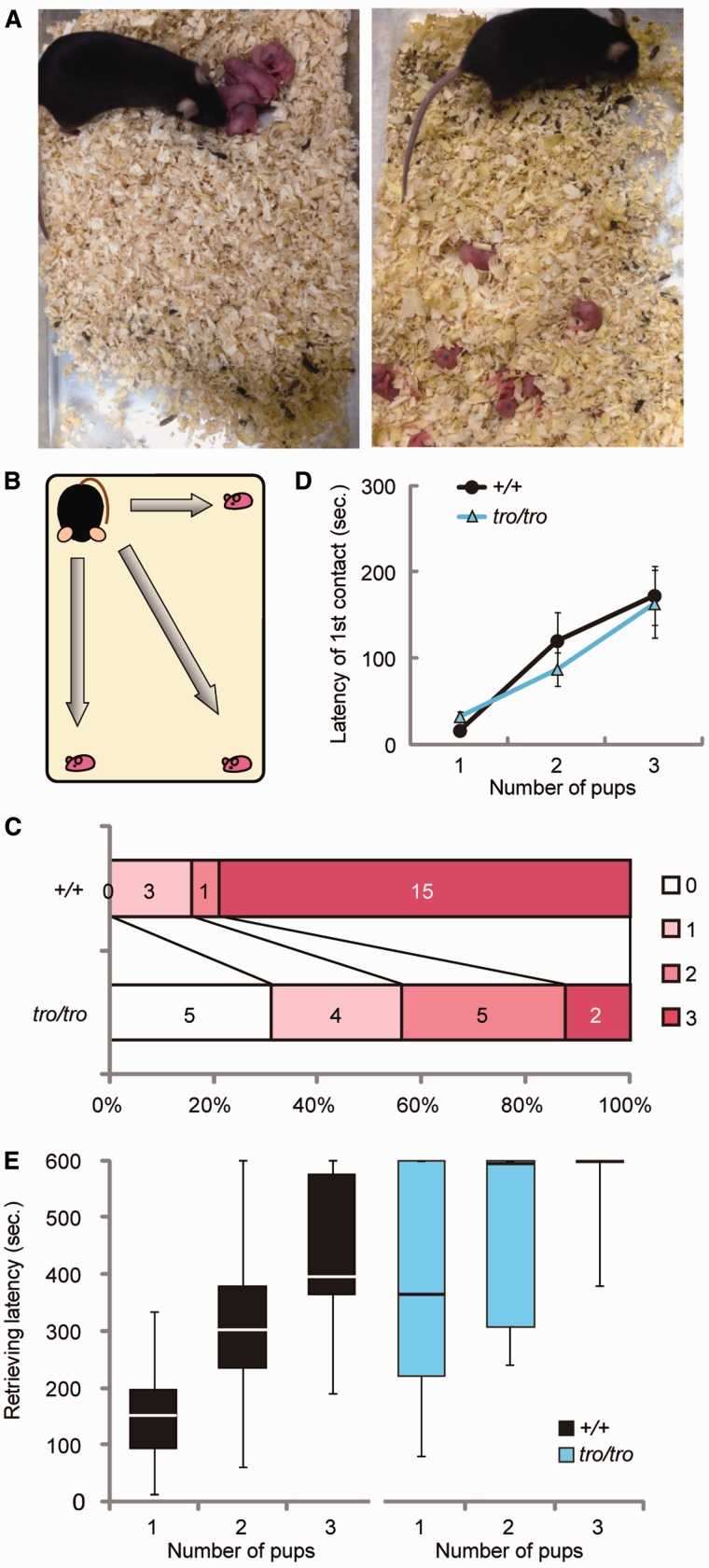
Retrieval test. (*A*) Example of the state in which females can retrieve and cannot retrieve. (*B*) Schematic of the test. (*C*) Distribution of the number of retrieved pups. Percentages and numbers of tested mice retrieving 3, 2, 1, or 0 pups are shown in each bar. (*D*) Latency to first contact in the retrieval test. Values represent mean ± SEM. (*E*) Box plot of retrieval latency for each pup. Top and bottom edge of box show the first and third quantile, respectively. Line in the box indicates median value. Top and bottom edge of line indicate maximum and minimum, respectively. *n* = 19 for *+/+* and *n* = 16 for *tro/tro*.

Mice exhibit a genetically programmed continuum of maternal behaviors from delivery to weaning, comprising nest building, pup retrieval, crouching on pups, placentophagia, licking, and lactation/nursing (Rosenblatt 1967). These behaviors are essential for pup survival, especially in the first several days after birth. Pup retrieval (returning pups to the nest by carrying them in the mouth) is the most important maternal behavior for most mammals because the pups cannot locomote immediately after birth and it is crucial for pups to maintain their body temperatures through nursing from dams (Lucas et al. 1998; Alenina et al. 2009). The *+/tro* and *tro/tro* dams showed no apparent defect in maternal behavior other than pup retrieval. We frequently observed that the pups were scattered in the cage (fig. 3*A*), although the *+/tro* and *tro/tro* dams had built the nest. Therefore, we performed a retrieval test using female mice (Lonstein and Fleming 2002; Jin et al. 2007). In the retrieval test, virgin female mice and three newborn *+/+* babies born to other mothers were used. These conditions were used because it was difficult to maintain experimental consistency such as for weekly age and number of deliveries when using dams and their biological pups. More importantly, the weaning ratio of pups from *tro/tro* dams was very low and it was thus difficult to furnish three biological pups for this test. The three pups were placed into each corner of a home cage in which a virgin female mouse built a nest in the fourth corner (fig. 3*B*). We recorded the contact latency (time until contact), retrieval latency, and number of retrieved pups. All of the *+/+* female mice contacted the pups, took them in their mouths, and retrieved at least one pup. Approximately 80% of the *+/+* female mice retrieved all three pups (fig. 3*C*). The *tro/tro* female mice also contacted all the pups, and the latency of the first contact with each pup did not differ from that of the *+/+* female mice (fig. 3*D*). Of the 16 *tro/tro* female mice tested, only two retrieved all three pups (12.5%) (fig. 3*C*). In addition, more time was necessary for *tro/tro* females to retrieve the pups (fig. 3*E*), and 5 of the 16 *tro/tro* females (31.2%) were not able to retrieve at all within 10 min of beginning the test (fig. 3*C*). Therefore, significantly fewer pups were retrieved by *tro/tro* females than by *+/+* females (*+/+* = 2.63, *tro/tro* = 1.25, *P* < 0.001), and *tro/tro* females had a longer retrieving latency than *+/+* females.

### Less Curiosity Toward Others and Diminished Recognition

**Fig. 4.—:**
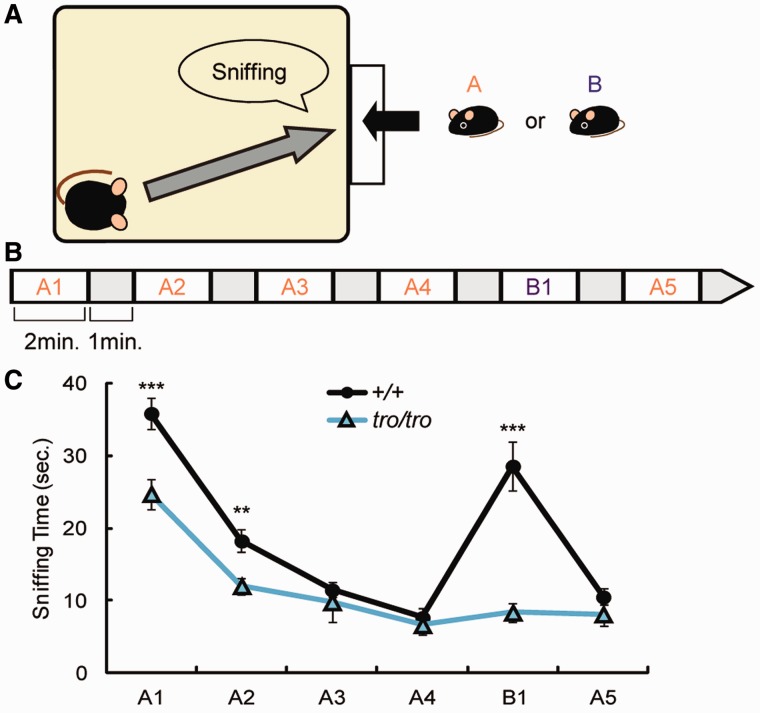
Habituation–dishabituation test. (*A*) Schematic of habituation–dishabituation test. (*B*) Paradigm and sequence for presenting the young mouse A or B. Each interval is 1 min. (*C*) Total sniffing time at each section. Values represent mean ± SEM. **P < 0.01, ***P < 0.001 (Student’s *t*-test).

Recognition of pups is important for retrieval (Lucas et al. 1998). To analyze the ability to recognize the pups, we performed a habituation–dishabituation test. In this test, a young *+/+* mouse A or B was presented to an adult virgin mouse and the time the adult mouse spent sniffing the young mouse was recorded (fig. 4*A*). When young mouse A was continuously presented to an adult *+/+* mouse four times (A1–A4), the time spent sniffing decreased gradually due to habituation. After the fourth presentation of young mouse A (A4), young mouse B was presented. The time spent sniffing increased compared with that of trial A4. Upon representation of young mouse A after young mouse B (A5), the amount of time spent sniffing again decreased, nearly equal to that in trial A4 (fig. 4*B*). These findings indicate that *+/+* mouse recognized that young mouse A and young mouse B were different individuals. In contrast, the sniffing pattern of the *tro/tro* mice was completely different. The *tro/tro* mice spent significantly less time sniffing than *+/+* mice in trials A1 and A2 (fig. 4*C*; *P* < 0.001 and *P* < 0.01 for A1 and A2, respectively). In the remaining trials (A3, A4, and A5), *tro/tro* mouse tended to spend less time sniffing than *+/+* mouse, although the difference was not significant. This finding indicates that *tro/tro* mice had less curiosity toward others. Moreover, the sniffing time in trial B1 was nearly equal to that of trial A4, indicating that *tro/tro* mice could not discriminate between young mouse A and young mouse B. Accordingly, there were no differences in the sniffing time among A4, B1, and A5 (fig. 4*C*). Olfaction is critical for social interactions in mammals: the ability to distinguish between offspring and nonoffspring is essential for the preferential investment of biological parents and prevention of the deleterious consequences of inbreeding (Potts et al. 1991; Yamazaki et al. 2000). Curiosity toward others, however, rather than olfactory recognition, seems to be significantly decreased in the *tro/tro* female mouse. Maternal neglect is not caused by disturbed olfaction in mice with knockout of *Tph2*, a rate-limiting enzyme of serotonin (5-HT) synthesis in brain (Alenina et al. 2009), whereas mice lacking most serotonergic neurons exhibit a drastic impairment in maternal care (Lerch-Haner et al. 2008). *Pou3f2* transactivates *Tph2* expression (Scheuch et al. 2007). Thus, monoamine neurotransmitters might have a key role in the impaired maternal behavior of the *Pou3f2* nonmammalized mice.

### Repeat Loss Leads to Diminished Pup Retrieval

To examine whether defects of retrieval behavior in nonmammalized *xPou3f2* knock-in mice are due to the lack of homopolymeric amino acid repeats unique to mammalian *Pou3f2*, we next investigated the knock-in mouse *Pou3f2 ΔGQP* knock-in, in which all of the three homopolymeric amino acid repeats of the murine *Pou3f2* gene were deleted. As well as appearance, body weight, growth, or reproductivity, there was no difference in expression level of the *Pou3f2* gene between wild-type and *ΔGQP* knock-in mice, like *xPou3f2* knock-in mice (supplementary fig. S3*A*, Supplementary Material online). Mating between heterozygous (*+/Δ*) male and female mice generated three genotypes of pups in accordance with Mendel’s laws (*+/+*:*+/Δ*:*Δ/Δ* = 81:153:77, *P* = 0.996, supplementary fig. S3*B*, Supplementary Material online). Histological analysis revealed no apparent brain abnormalities in *Δ/Δ* mice. Nevertheless, the number of weaned pups from *Δ/Δ* dams was significantly lower. To examine the genotype of dams or pups responsible for the failed weaning, we compared the weaning ratio of pups born from the following matings: 1) *+/+* dam and *+/+* sire, 2) *+/+* dam and *Δ/Δ* sire, 3) *Δ/Δ* dam and *+/+* sire, and 4) *Δ/Δ* dam and *Δ/Δ* sire (supplementary fig. S3*C*, Supplementary Material online). The statistical analysis clearly indicated that 1) the decreased weaning ratio of pups was dependent on the genotype of the dams and 2) the genotypes of the sires and pups were unrelated to the decreased weaning ratio of the pups. Like *tro/tro* dams, *Δ/Δ* dams showed no apparent defects in maternal behavior other than pup retrieval. Pups were frequently observed to be scattered in the cage. In the retrieval test, the latency to the first contact did not differ between *+/+* and *Δ/Δ* female mice. There was also no difference in the latency to the first contact with the second and third pups. The number of females that retrieved all three pups was 11 of 17 (68.8%) and 2 of 19 (10.5%) for the *+/+* and *Δ/Δ* mice, respectively. Consequently, *+/+* and *Δ/Δ* females retrieved 2.25 or 1.13 pups on average, respectively (*P* < 0.05).

*Pou3f2 ΔGQP* knock-in female mice with complete deletion of the three homopolymeric amino acid repeats of the *Pou3f2* gene, and *xPou3f2* knock-in female mice in which the entire coding region of the *Pou3f2* gene was replaced with that of the *X. tropicalis* ortholog missing all three homopolymeric amino acid repeats exhibited the same behavior: remarkably low weaning ratio of their pups representative of defective maternal behaviors and drastically decreased pup retrieval. This finding implies that the homopolymeric amino acid repeats of mammalian POU3F2 play an important role in mammalian maternal behavior.

### Declining Monoaminergic Function

Dopamine (DA) is a neurotransmitter in the brain with vital roles in a variety of different behaviors. The major behaviors affected by DA are movement, cognition, pleasure, and motivation, as well as a number of basic functions, including lactation and nausea (Kandel et al. 2013). Dopamine is classified as a catecholamine synthesized from tyrosine by TH, a rate-limiting enzyme. High concentrations of DA and TH are localized in the neostriatum, nucleus accumbens, olfactory tubercle, and hypothalamus, together with other some regions (Sutoo et al. 2002; Kandel et al. 2013). Serotonin is another a neurotransmitter in the brain involved in a wide variety of brain functions, such as mood control, regulation of sleep and body temperature, anxiety, drug abuse, food intake, and sexual behavior (Walther and Bader 2003). Serotonergic neuronal cell bodies are located primarily in the raphe nucleus and these cells project throughout the brain where 5-HT contributes to a variety of different behavioral and physiological functions (Kandel et al. 2013). Both TH and TPH2 are widely used as markers for dopaminergic neurons and serotonergic neurons, respectively, in the brain to investigate various brain functions and pathogenic mechanisms of neurodegenerative diseases or psychiatric disorders.

To elucidate the brain mechanisms of behavioral abnormalities, including abnormal maternal behavior, we first compared the immunohistochemical distribution of both enzymes in the brains of these knock-in mice using laser confocal microscopy. There were clear differences in TH and TPH2 between the brains of *+/+* mice and *tro/tro* or *Δ/Δ* mice (supplementary fig. S4, Supplementary Material online). We conducted detailed analyses of both enzymes in the brain using a quantitative fluorescence microphotometry system, MapAnalyzer (Sutoo et al. 1998).

**Fig. 5.—:**
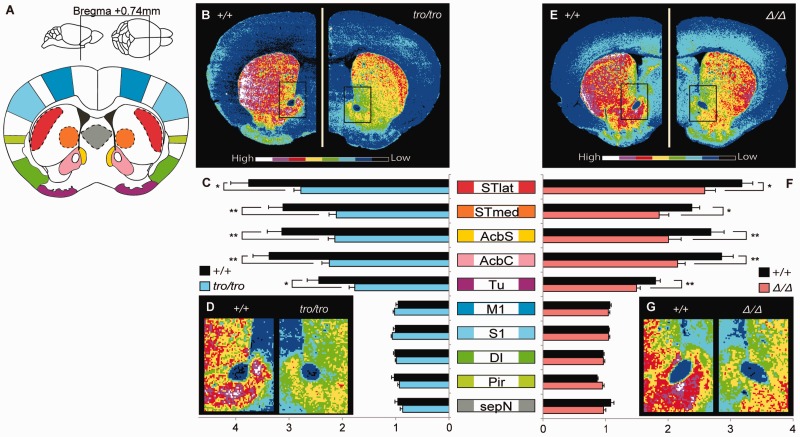
Quantitative immunohistochemistry for TH. (*A*) Fluorescence intensities were measured in transverse sections located approximately 0.74 mm rostral from bregma, including DA-rich regions, the neostriatum, and nucleus accumbens. Abbreviations (Franklin and Paxinos 2008): STlat, lateral part of the neostriatum; STmed, medial part of the neostriatum; AcbS, shell of the nucleus accumbens; AcbC, core of the nucleus accumbens; Tu, olfactory tubercle; M1, primary motor cortex; S1, primary sensory cortex; DI, dysgranular insular cortex; Pir, piriform cortex; and sepN, septal area. (*B–D*) Quantitative distribution, comparative analysis, and enlarged ventral striatum region for *+/+* and *tro/tro*, respectively. (*E–G*) Quantitative distribution, comparative analysis, and enlarged ventral striatum region for *+/+* and *Δ/Δ*, respectively. Values shown on graphs represent the mean ± SEM. *P < 0.05; **P < 0.01.

The immunohistochemical distributions of TH in the brain are shown in figure 5*A*–*D*. The highest fluorescence intensity in *+/+* female mice was observed in the neostriatum and nucleus accumbens, followed by the olfactory tubercle; the cerebral cortex and septal area had low levels. In contrast to the *+/+* mice, the fluorescence intensities of TH in *tro/tro* female mice were significantly lower in the lateral (26%, *P* < 0.05) and medial (32%, *P* < 0.01) parts of the neostriatum, the shell (32%, *P* < 0.01) and core (34%, *P* < 0.01) of the nucleus accumbens, and the olfactory tubercle (28%, *P* < 0.05). There were changes in the reduction ratio within the neostriatum and the nucleus accumbens; the reduction ratios of TH in the medial part of the neostriatum, and in the shell and core of the nucleus accumbens were greater than that in lateral part of the neostriatum. The TH levels in the cerebral cortex and septal area of *tro/tro* mice were not significantly different from those in *+/+* mice.

The relationship between DA in the brain and maternal behavior has been widely investigated. Injection of a D_1_ or D_2_ receptor antagonist into the neostriatum or nucleus accumbens of postpartum rats disrupts pup retrieval, pup licking, or nest building (Keer and Stern 1999; Numan et al. 2005; Zhao and Li 2010). Lactating rats injected with the catecholaminergic neurotoxin 6-hydroxydopamine in the ventral striatum perform poorly in pup retrieval tests (Hansen et al. 1991). In addition, mice genetically depleted of DA from all striatal areas exhibit severe deficits in pup retrieval and licking/grooming behavior (Henschen et al. 2013). There is a significant rise in the extracellular concentrations of DA in the ventral striatum and nursing behavior is predominant when mother rats are reunited with their litters following overnight separation (Hansen et al. 1993).

**Fig. 6.—:**
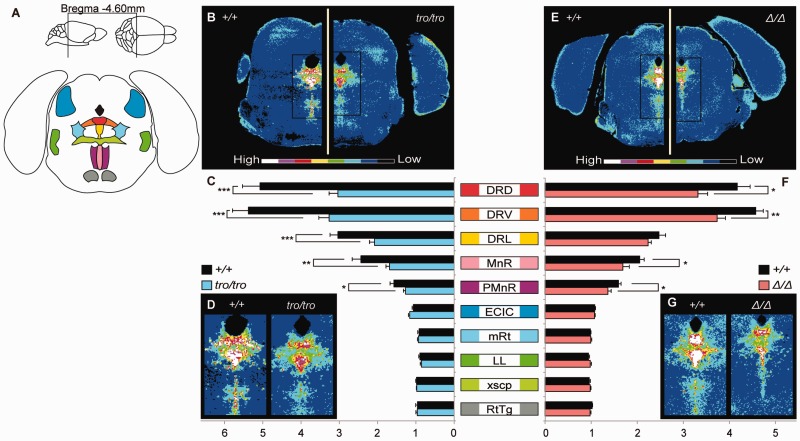
Quantitative immunohistochemistry for TPH2. (*A*) Fluorescence intensities were measured in transverse sections located approximately 4.60 mm caudal from bregma, including 5-HT-rich region, the raphe nucleus. Abbreviations (Franklin and Paxinos 2008): DRD, dorsal part of the dorsal raphe nucleus; DRV, ventral part of the dorsal raphe nucleus; DRL, lateral part of the dorsal raphe nucleus; MnR, median raphe nucleus; PMnR, paramedian raphe nucleus; ECIC, external cortex of the inferior colliculus; mRt, mesencephalic reticular formation; LL, lateral lemniscus; xscp, decussation of the superior cerebellar peduncle; RtTg, reticulotegmental nucleus of the pons. (*B–D*) Quantitative distribution, comparative analysis, and enlarged the raphe nucleus for *+/+* and *tro/tro*, respectively. (*E–G*) Quantitative distribution, comparative analysis, and enlarged the raphe nucleus for *+/+* and *Δ/Δ*, respectively. Values shown on graphs represent the mean ± SEM. *P < 0.05; **P < 0.01; ***P < 0.001.

In addition to TH, the distributions of TPH2 are shown in figure 6*A*–*D*. TPH2 levels in *+/+* female mice were highest in the dorsal raphe nucleus followed by the median raphe nucleus and paramedian raphe nucleus. TPH2 levels were low in the external cortex of the inferior colliculus, lateral lemniscus, reticulotegmental nucleus of the pons, mesencephalic reticular formation, and the decussation of the superior cerebellar peduncle. In contrast to *+/+* mice, the fluorescence intensities of TPH2 in *tro/tro* female mice were significantly decreased in the dorsal (40%, *P* < 0.001), ventral (39%, *P* < 0.001), and lateral (31%, *P* < 0.001) parts of the dorsal raphe nucleus; the median raphe nucleus (31%, *P* < 0.01); and the paramedian raphe nucleus (19%, *P* < 0.05). The TPH2 levels in other brain regions of *tro/tro* mice were not significantly different compared with those in *+/+* mice.

Brain 5-HT affects anxiety and impulsivity, which in turn may affect maternal behaviors such as infant retrieval or rejection of infants’ attempts to make contact with the dam (Saltzman and Maestripieri 2011). Rats with selective lesions of serotonergic neurons in the median raphe nucleus exhibit a higher incidence of failure to retrieve pups and initiate suckling (Barofsky et al. 1983). Also, selective blocking of 5-HT receptors disrupts pup retrieval and nest building (Zhao and Li 2010). *Tryptophan hydroylase 2* knockout female mice that lack 5-HT in the central nervous system, despite being fertile and producing milk, exhibit impaired maternal care leading to poor survival of their pups (Alenina et al. 2009). Serotonin_1A_ receptor-null dams exhibit impaired pup retrieval and nest building, leading to reduced weight gain in their offspring (van Velzen and Toth 2010). Transgenic mouse dams with a specific disruption in 5-HT neuron development display profound maternal deficits (Lerch-Haner et al. 2008).

Based on these previous findings, we suggest that DA in the neostriatum, nucleus accumbens, and olfactory tubercle, as well as 5-HT in the raphe nucleus have important roles in maternal behavior, and that abnormal behavior in our knock-in mice originated from a decline in dopaminergic and serotonergic functions in these brain regions. The change in TH in the nucleus accumbens and medial part of the neostriatum of *tro/tro* mice was greater than that in the lateral part of the neostriatum. The nucleus accumbens, olfactory tubercle, and ventromedial caudate collectively form the ventral striatum (Björklund and Dunnett 2007). Maternal behavior is profoundly associated with the mesolimbic dopaminergic system, which projects from DA cell bodies in the ventral tegmental area to the ventral striatum (Numan and Stolzenberg 2009) and is a major component of the brain reward system (Robbins and Everitt 1996). Dysfunction of the reward system causes anhedonia (Keller et al. 2013), a core symptom of major depression, and may disrupt maternal behavior.

Immunohistochemical levels of TH in the neostriatum, nucleus accumbens, and olfactory tubercle, and of TPH2 in the raphe nucleus were decreased significantly in *Δ/Δ* mice compared with *+/+* mice, similar to *tro/tro* mice (figs. 5*E*–*G* and 6 *E*–*G*). The fluorescence intensities of TH in *Δ/Δ* female mice were significantly decreased in the lateral (19%, *P* < 0.05) and medial (22%, *P* < 0.05) parts of the neostriatum, the shell (25%, *P* < 0.05) and core (25%, *P* < 0.01) of the nucleus accumbens, and the olfactory tubercle (17%, *P* < 0.01) compared with those in *+/+* mice. The fluorescence intensities of TPH2 in *Δ/Δ* female mice were significantly decreased in the dorsal (21%, *P* < 0.05) and ventral (18%, *P* < 0.01) parts of the dorsal raphe nucleus, the median raphe nucleus (18%, *P* < 0.05), and the paramedian raphe nucleus (14%, *P* < 0.05) compared with *+/+* mice.

**Fig. 7.—:**
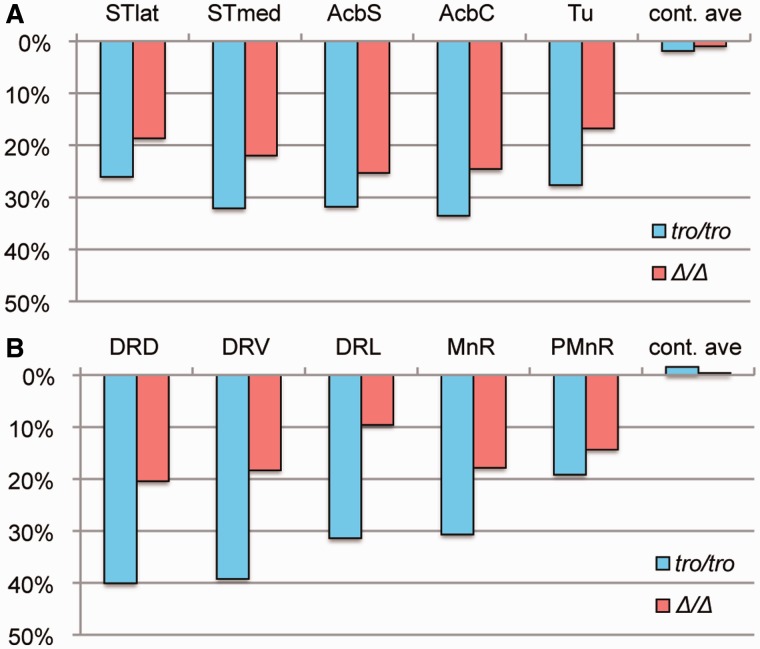
Reduction rates of TH and TPH2. (*A*) Reduction rate of TH in five regions (STlat, STmed, AcbS, AcbC, and Tu). “cont.ave” shows average of cortex regions (M1, S1, DI, and Pir). (*B*) Reduction rate of TPH2 in five regions (DRD, DRV, DRL, MnR, and PMnR). “cont.ave” shows average of other regions (ECIC, mRt, LL, xscp, and RtTg). Reduction rates were calculated as percentage of *tro/tro* (blue bars) or *Δ/Δ* (red bars) against *+/+*.

The ratios of TH and TPH2 in *tro/tro* to *+/+* mice were lower than those of *Δ/Δ* to *+/+* mice (fig. 7). One possible reason for this is that *tro/tro* displayed more prominent phenotypes is amino acid substitutions in DNA-binding domains. This possibility can be excluded, however, because the amino acid sequences of the DNA-binding domains of xPOU3F2 are identical to those of mouse POU3F2. Another possible reason is amino acid substitutions in the transactivation domain (in addition to the absence of repeats) and accompanying conformational changes of the POU3F2 protein. The prediction of intrinsically disordered regions differed slightly among amino acid sequences of wild, *Δ*, and *tro* alleles (supplementary fig. S5, Supplementary Material online). Our discovery suggests that pup retrieval, an essential mammalian maternal behavior, is established via enhanced dopaminergic and serotonergic neurotransmission in the brain by homopolymeric amino acid repeats acquired in the mammalian POU3F2. Hundreds of mammalian genes have homopolymeric amino acid repeats, ∼80% of which are evolutionarily well conserved in length (Mularoni et al. 2006; Gojobori and Ueda 2011). The majority of genes having repeats play important roles in transcription/translation and signaling processes (Faux et al. 2005), but very little is known about the evolutionary implications of repeat acquisition. Nonmammalized knock-in mice are a promising approach toward gaining a deeper understanding of the traits that characterize mammals.

Dopamine and 5-HT are important for the regulation of various psychological and physiological functions, such as pleasant feelings, anxiety, fear, mood control, motivation, cognition, reward, movement, regulation of body temperature, and drug abuse, as well as maternal behavior. Based on the findings described above, we hypothesize that the regulation of these functions is not sufficiently strict in nonmammals, including amphibians. Although nonamniotes such as fish and amphibians behave almost instinctively, nonhominoid amniotes exhibit noninstinctive behaviors, namely emotion, social behaviors, and nurturing. We expected the knock-in mice in the present study to have emotion-related variations, and thus consider that *Pou3f2* is important for mammalian brain evolution resulting in complicated behaviors. Further studies are needed to analyze the associations among *Pou3f2* and other characteristic mammalian behaviors, such as learning, memory, and thinking.

## Supplementary Material

Supplementary figures S1–S5 and table S1 are available at *Genome Biology and Evolution* online (http://www.gbe.oxfordjournals.org/).

Supplementary Data
