# Supplementary material for: Mammalian-Specific Sequences in Pou3f2 Contribute to Maternal Behavior
Source: Genome Biol Evol. 2014 Apr 7;6(5):1145–56. doi: 10.1093/gbe/evu072 (PMC4040985; doi:10.1093/gbe/evu072)
Supplement: Supplementary Data [file supp_6_5_1145__index.html]

Mammalian-Specific Sequences in Pou3f2 Contribute to Maternal Behaviour — Mammalian-Specific Sequences in Pou3f2 Contribute to Maternal Behavior — Supplementary Data 

# Mammalian-Specific Sequences in *Pou3f2* Contribute to Maternal Behavior

## Supplementary Data

files

**Files in this Data Supplement:**

- Supplementary Data - pdf file
