# Supplementary material for: Mammalian-Specific Sequences in Pou3f2 Contribute to Maternal Behavior
Source: Genome Biol Evol. 2014 Apr 7;6(5):1145–56. doi: 10.1093/gbe/evu072 (PMC4040985; doi:10.1093/gbe/evu072)
Supplement: Supplementary Data [file supp_evu072_GBE-140125_SI_rev_Unmarked.pdf]

Supplementary Material

Mammalian-Specific Sequences in *Pou3f2* Contribute to Maternal Behaviour

Makoto Nasu, Saori Yada, Atsushi Igarashi, Den'etsu Sutoo, Kayo Akiyama, Meguru Ito, Nobuaki Yoshida, and Shintaroh Ueda

Supplementary table and figure legends

Table S1

Sequence information of vertebrate species used in supplementary fig. S1.

Species	Latin name	Transcript ID
Human	*Homo sapiens*	NM 005604.2
Chimpanzee	*Pan troglodytes*	AB554076
Gorilla	*Gorilla gorilla*	AB554075
Orangutan	*Pongo pygmaeus*	AB554074
Cow	*Bos taurus*	NM 001080348.1/ENSBTAT00000034488
Lesser hedgehog tenrec	*Echinops telfairi*	ENSETET00000020341
Mouse	*Mus musculus*	NM 008899.1/ENSMUST00000064587
Rat	*Rattus norvegicus*	NM 172085.1/ENSRNOT00000009043
Opossum	*Monodelphis domestica*	ENSMODT00000022991
Japanese quail	*Coturnix japonica*	AF091043.1
Green anole	*Anolis carolinensis*	AB001869.1
Xenopus laevis	*Xenopus laevis*	NM 001101755.1
Xenopus tropicalis	*Xenopus tropicalis*	AB557604
Medaka	*Oryzias latipes*	ENSORLT00000002764
Stickleback	*Gasterosteus aculeatus*	ENSGACT00000006578
Takifugu	*Takifugu rubripes*	ENSTRUT00000038615
Tetrodon	*Tetraodon nigroviridis*	ENSTNIT00000016300
Zebrafish	*Danio renio*	NM 131160.1/ENSDART00000113914

Figure S1.

Multiple alignment of POU3F2s among *Vertebrata.*

For multiple alignment, we used MAFFT on a CLC Bio Sequence Viewer. Sequence data contained nine mammals, one bird, one reptile, two amphibians, and five fish (See supplementary table S1). Red boxes indicate homopolymeric amino acid repeats, polyG, polyQ, and polyP. Blue boxes indicate DNA binding domain, POU-specific domain, and POU-homeodomain. Sequence gaps are indicated by the hyphen.

Figure S2.

Comparison between +/+ and *tro/tro* mice.

(A) Immunohistochemical distribution of POU3F2.

(B) Body weight.

(C) Brain weight per body weight.

(D) Nissl staining of brain coronal section around hypothalamus.

(E) Nissl staining of brain sagittal section.

Abbreviations: Cx, cerebral cortex; PVN, paraventricular nucleus; SO, supraoptic nucleus; DG, dentate gyrus; CA1, cornet d'Ammon; ST, neostriatum; DR, dorsal raphe nucleus; SCh, suprachiasmatic nucleus; 3V, third ventricle; LV, lateral ventricle; 4V, fourth ventricle.

Figure S3.

Comparison among +/+, +/*Δ*, and *Δ/Δ* mice.

(A) Relative expression level of *Pou3f2* by qPCR.

Each data is indicated as a ratio to the expression level of +/+ mice. No statistical differences were observed among +/+, +/*Δ*, and *Δ/Δ* mice.

(B) Genotype ratio of pups from +/*Δ* parents.

(C) Pups viability. Black, meshed red, and red bar represent +/+, +/*Δ*, and *Δ/Δ*, respectively. $^{**}P<0.01$.

Figure S4.

Confocal images of tryptophan hydroxylase 2 immunohistochemistry in the raphe nucleus.

(A) +/+ (left) and *tro/tro* mice (right) in the raphe nucleus.

(B) +/+ (left) and *Δ/Δ* mice (right) in the raphe nucleus.

(C) Atlas of the raphe nucleus.

Abbreviations: Aq, aqueduct; DRD, dorsal part of the dorsal raphe nucleus; DRV, ventral

part of the dorsal raphe nucleus; DRL, lateral part of the dorsal raphe nucleus.

Figure S5.

Predicted profile of the intrinsically disordered structure of mouse POU3F2, mouse POU3F2 *Δ*GQP, and *Xenopus tropicalis* POU3F2.

This analysis was performed on the PrDOS server to predict intrinsically disordered regions of the protein from the amino acid sequence (http://prdos.hgc.jp/cgi-bin/top.cgi/) (Ishida and Kinoshita 2007). Many proteins have regions with very flexible and unstable structures, referred to as intrinsically disordered, even in their native states. Disordered regions are involved in many biological processes, such as regulation, signalling, and cell cycle control. The primary role of intrinsically disordered regions seems to be the molecular recognition of proteins or DNA. Upon binding with ligands, disorder-to-order transitions are frequently observed, where the flexibility of disordered regions may be necessary to interact with multiple partners with high-specificity and low-affinity. Therefore, the prediction of disordered regions in proteins is important for the functional annotation of proteins. PrDOS: prediction of disordered protein regions from amino acid sequence.

(A-C) Transition of sequential disordered probability of amino acid sequences; mouse POU3F2 (A), POU3F2 *Δ*GQP form (B), and *X. tropicalis* POU3F2 (C). Prediction false positive rate was set to 5.0%. Orange boxes indicate homopolymeric amino acid repeats and orange lines indicate the positions where homopolymeric amino acid repeats are inserted in mammals. Blue boxes represent POU-specific domain and POU-homeodomain.

(D-F) Properties of disordered probability in sequences. Amino acids with high disordered probability (>0.5 threshold) are highlighted in red. Yellow boxes indicate homopolymeric amino acid repeats and yellow lines indicate the positions where homopolymeric amino acid repeats are inserted in mammals. Blue boxes represent POU-specific domain and POU-homeodomain.

In POU3F2, most of the homopolymeric amino acid repeats showed high disordered probability. Therefore, disordered values of the sequence without homopolymeric amino acid repeats were comparatively low in the respective regions in POU3F2 *Δ*GQP and xPOU3F2. Moreover, the disordered probability of N-terminal 65 residues of xPOU3F2 is low in comparison with mouse POU3F2 and POU3F2 *Δ*GQP, in which homopolymeric glycine repeats are uniquely present in mammals. Therefore, this difference between POU3F2 *Δ*GQP and xPOU3F2 might cause prominent phenotype differences in behavioural tests and monoamine neurotransmission between *tro/tro* and *Δ/Δ* mice. In mammalian

POU3F2, the acquisition of not only homopolymeric amino acid repeats but also amino acid substitutions in transactivation domains might contribute to mammal-specific function and expression.

Reference

Ishida T, Kinoshita K. 2007. PrDOS: prediction of disordered protein regions from amino acid sequence. Nucleic Acids Res. 35: W460-W464.

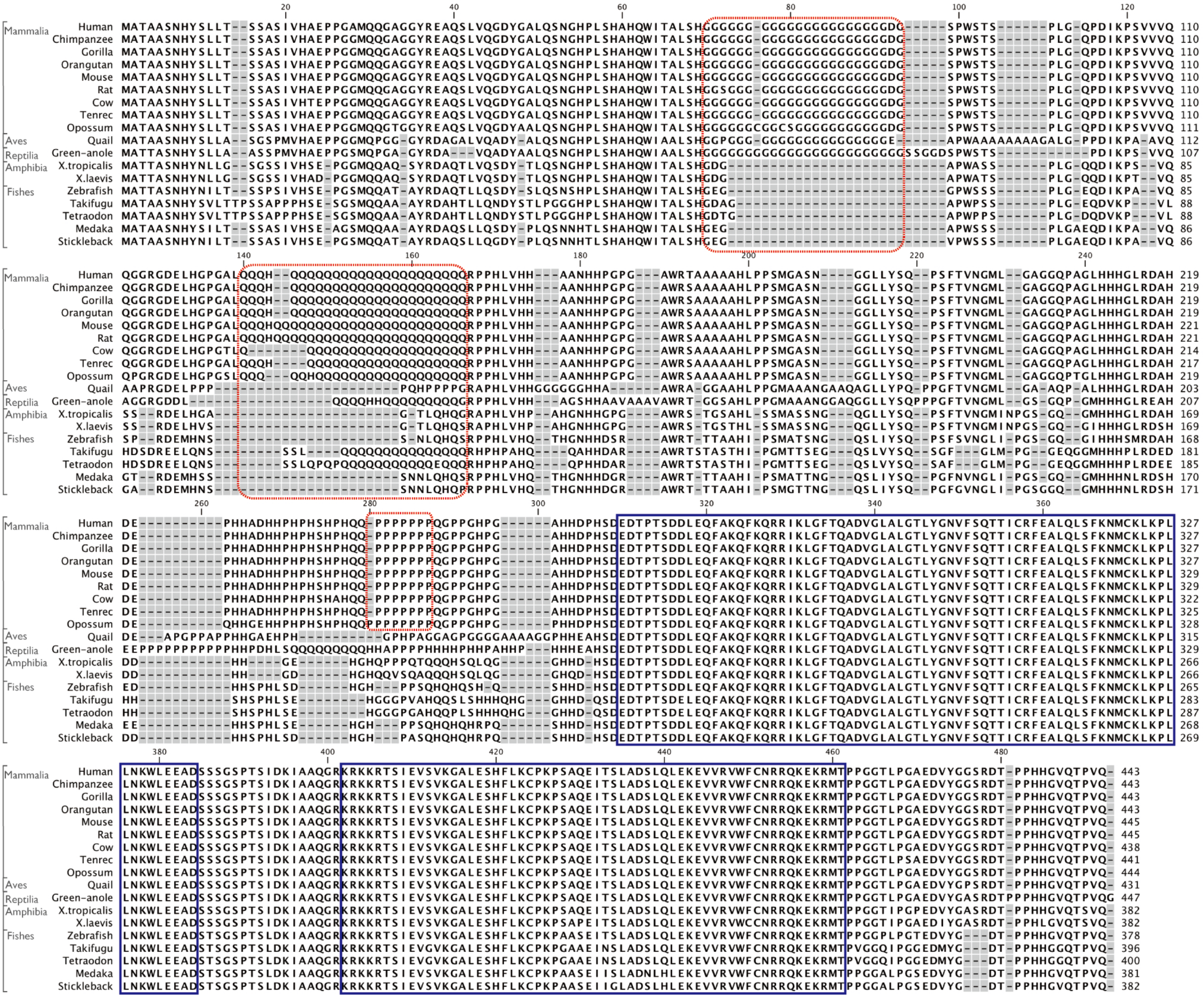
Mammalia
Human
Chimpanzee
Gorilla
Orangutan
Mouse
Rat
Cow
Tenrec
Opossum
Aves
Quail
Reptilia
Green-anole
Amphibia
X.tropicalis
X.laevis
Fishes
Zebrafish
Takifugu
Tetraodon
Medaka
Stickleback

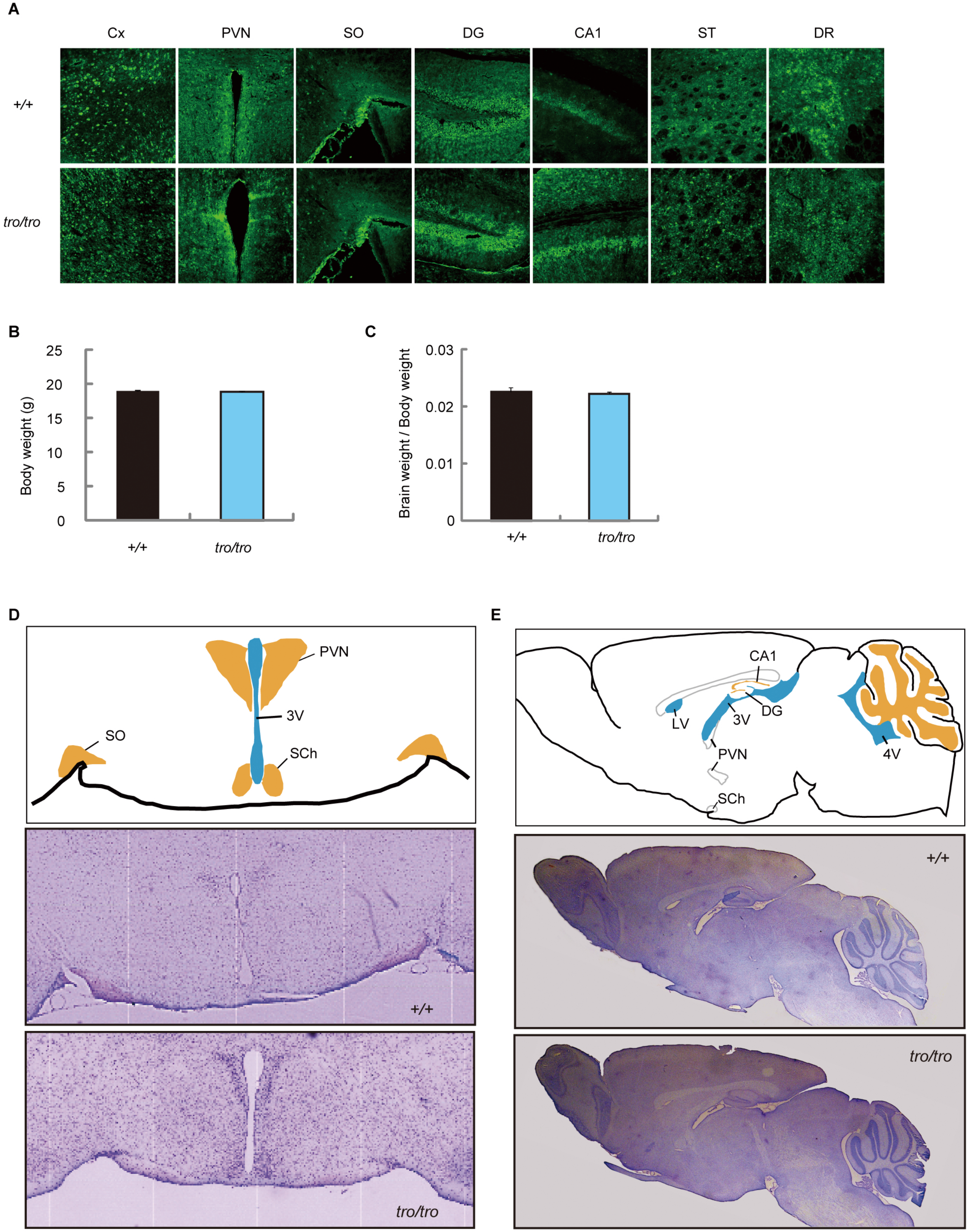

A
Cx
PVN
SO
DG
CA1
ST
DR
+/+
tro/tro
B
Body weight (g)
25
20
15
10
5
0
+/+
tro/tro
C
Brain weight / Body weight
0.03
0.02
0.01
0
+/+
tro/tro
D
PVN
3V
SO
SCh
+/+
tro/tro
E
CA1
LV
3V
DG
PVN
SCh
4V
+/+
tro/tro

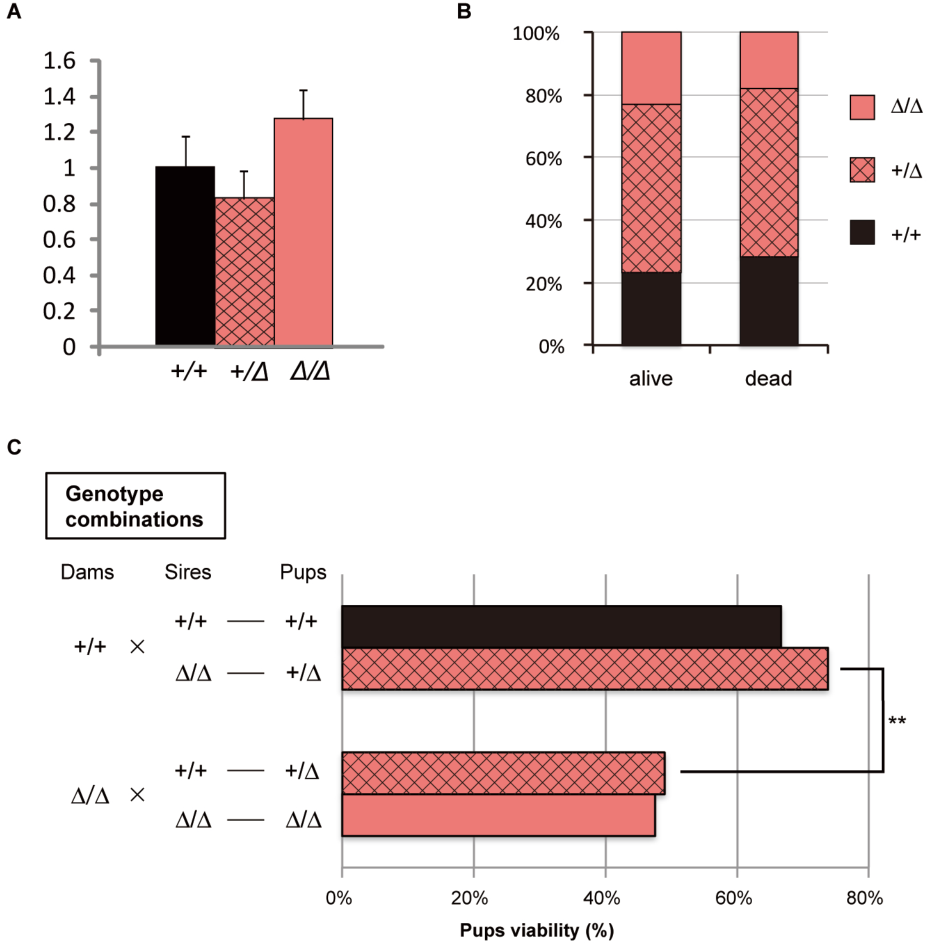
A
1.6
1.4
1.2
1
0.8
0.6
0.4
0.2
0
+/+
+/Δ
Δ/Δ
B
100%
80%
60%
40%
20%
0%
alive
dead
Δ/Δ
+/Δ
+/+
C
Genotype combinations
Dams
Sires
Pups
+/+ ×
+/+ — +/+
Δ/Δ — +/Δ
Δ/Δ ×
+/+ — +/Δ
Δ/Δ — Δ/Δ
**
0%
20%
40%
60%
80%
Pups viability (%)

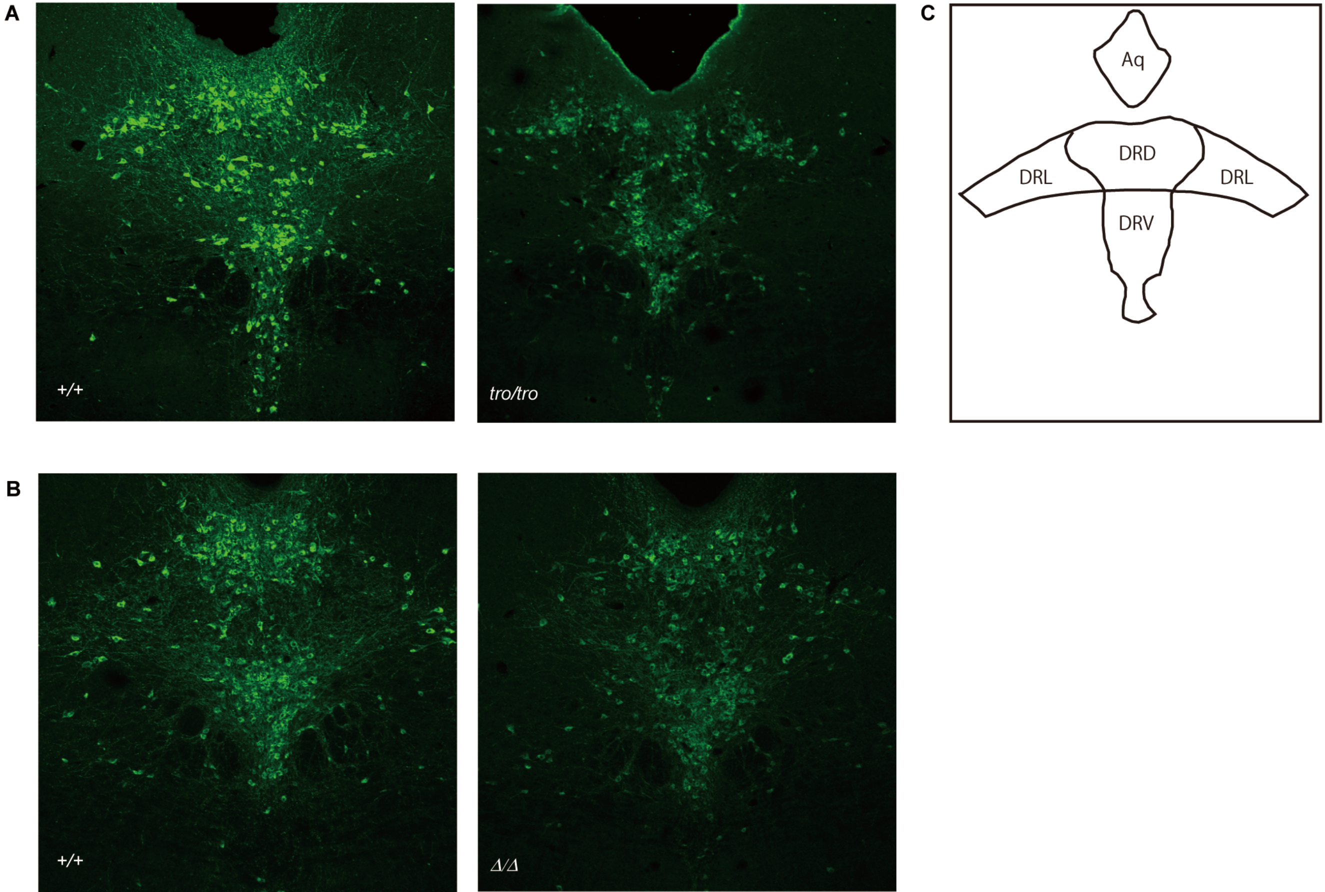
A
+/+
tro/tro
B
+/+
Δ/Δ
C
Aq
DRD
DRL
DRL
DRV

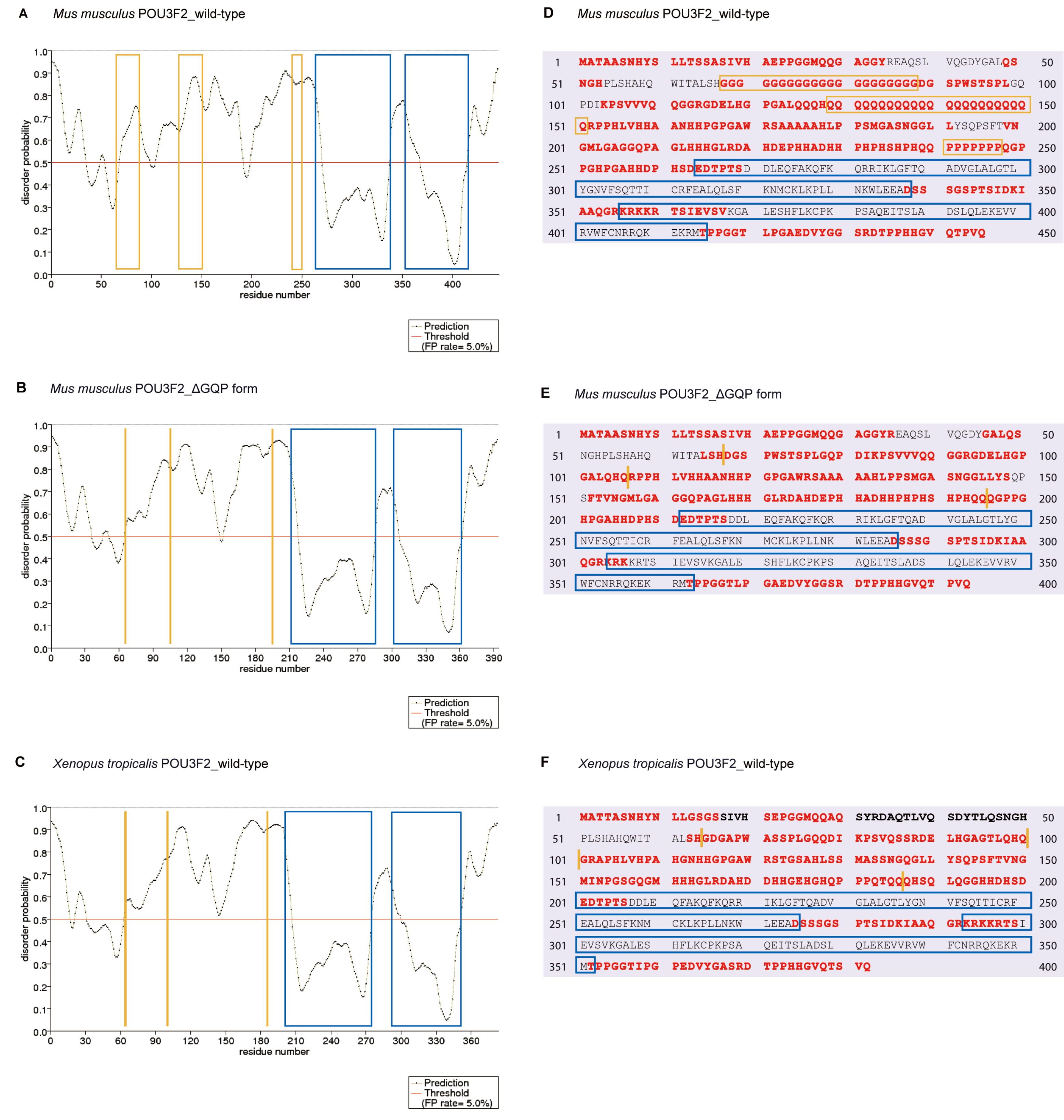
A
Mus musculus POU3F2_wild-type
disorder probability
residue number
Prediction
Threshold
(FP rate= 5.0%)
B
Mus musculus POU3F2_ΔGQP form
disorder probability
residue number
Prediction
Threshold
(FP rate= 5.0%)
C
Xenopus tropicalis POU3F2_wild-type
disorder probability
residue number
Prediction
Threshold
(FP rate= 5.0%)
D
Mus musculus POU3F2_wild-type
1 MATAASNHYS LLTSSASIVH AEPPGGMQQG AGGYREAQSL VQGDYGALQS 50
51 NGHPLSHAHQ WITALSHGGG GGGGGGGGGG GGGGGGGGDG SPWSTSPLGQ 100
101 PDIKPSVVVQ QGGRGDELHG PGALQQHQQ QQQQQQQQQQ QQQQQQQQQQ 150
151 QRPPHLVHHA ANHHPGPGAW RSAAAAAHLP PSMGASNGGL LYSQPSFTVN 200
201 GMLGAGGQPA GLHHHGLRDA HDEPHHADHH PHPHSHPHQQ PPPPPPPQGP 250
251 PGHPGAHHDP HSDEDTPTSD DLEQFAKQFK QRRIKLGFTQ ADVGLALGTL 300
301 YGNVFSQTTI CRFEALQLSF KNMCKLKPLL NKWLEEADSS SGSPTSIDKI 350
351 AAQGRKRKKR TSIEVSVKGA LESHFLKCPK PSAQEITSLA DSLQLEKEVV 400
401 RVWFCNRRQK EKRMTPPGGT LPGAEDVYGG SRDTPPHHGV QTPVQ 450
E
Mus musculus POU3F2_ΔGQP form
1 MATAASNHYS LLTSSASIVH AEPPGGMQQG AGGYREAQSL VQGDYGALQS 50
51 NGHPLSHAHQ WITALSHDGS PWSTSPLGQP DIKPSVVVQQ GGRGDELHGP 100
101 GALQHQRPPH LVHHAANHHP GPGAWRSAAA AAHLPPSMGA SNGGLLYSQP 150
151 SFTVNGMLGA GGQPAGLHHH GLRDAHDEPH HADHHPHPHS HPHQQQGPPG 200
201 HPGAHHDPHS DEDTPTSDDL EQFAKQFKQR RIKLGFTQAD VGLALGTLYG 250
251 NVFSQTTICR FEALQLSFKN MCKLKPLLNK WLEEADSSSG SPTSIDKIAA 300
301 QGRKRKKRTS IEVSVKGALE SHFLKCPKPS AQEITSLADS LQLEKEVVRV 350
351 WFCNRRQKEK RMTPPGGTLP GAEDVYGGSR DTPPHHGVQT PVQ 400
F
Xenopus tropicalis POU3F2_wild-type
1 MATTASNHYN LLGSGSSIVH SEPGGMQQAQ SYRDAQTLVQ SDYTLQSNGH 50
51 PLSHAHQWIT ALSHGDGAPW ASSPLGQQDI KPSVQSSRDE LHGAGTLQHQ 100
101 GRAPHLVHPA HGNHHGPGAW RSTGSAHLSS MASSNGQGLL YSQPSFTVNG 150
151 MINPGSGQGM HHHGLRDAHD DHHGEHGHQP PPQTQQQHSQ LQGGHHDHSD 200
201 EDTPTSDDLE QFAKQFKQRR IKLGFTQADV GLALGTLYGN VFSQTTICRF 250
251 EALQLSFKNM CKLKPLLNKW LEEADSSSGS PTSIDKIAAQ GRKRKKRTSI 300
301 EVSVKGALES HFLKCPKPSA QEITSLADSL QLEKEVVRVW FCNRRQKEKR 350
351 MTPPGGTIPG PEDVYGASRD TPPHHGVQTS VQ 400
